# Bacterial Biotransformation of Pentachlorophenol and Micropollutants Formed during Its Production Process

**DOI:** 10.3390/ijerph13111146

**Published:** 2016-11-17

**Authors:** Eglantina Lopez-Echartea, Tomas Macek, Katerina Demnerova, Ondrej Uhlik

**Affiliations:** Department of Biochemistry and Microbiology, Faculty of Food and Biochemical Technology, University of Chemistry and Technology, Prague 16628, Czech Republic; tomas.macek@vscht.cz (T.M.); demnerok@vscht.cz (K.D.)

**Keywords:** pentachlorophenol, polychlorinated dibenzo-*p*-dioxins, polychlorinated dibenzofurans, bioremediation, wood preservation, contaminated soil, bacterial degradation

## Abstract

Pentachlorophenol (PCP) is a toxic and persistent wood and cellulose preservative extensively used in the past decades. The production process of PCP generates polychlorinated dibenzo-*p*-dioxins and polychlorinated dibenzofurans (PCDD/Fs) as micropollutants. PCDD/Fs are also known to be very persistent and dangerous for human health and ecosystem functioning. Several physico-chemical and biological technologies have been used to remove PCP and PCDD/Fs from the environment. Bacterial degradation appears to be a cost-effective way of removing these contaminants from soil while causing little impact on the environment. Several bacteria that cometabolize or use these pollutants as their sole source of carbon have been isolated and characterized. This review summarizes current knowledge on the metabolic pathways of bacterial degradation of PCP and PCDD/Fs. PCP can be successfully degraded aerobically or anaerobically by bacteria. Highly chlorinated PCDD/Fs are more likely to be reductively dechlorinated, while less chlorinated PCDD/Fs are more prone to aerobic degradation. The biochemical and genetic basis of these pollutants’ degradation is also described. There are several documented studies of effective applications of bioremediation techniques for the removal of PCP and PCDD/Fs from soil and sediments. These findings suggest that biodegradation can occur and be applied to treat these contaminants.

## 1. Introduction

Pentachlorophenol (PCP) is an aromatic hydrocarbon of the chlorophenol (CP) family, first introduced as a wood preservative in the 1930s. Since introduced, PCP has had a variety of other applications, for instance as a biocide, pesticide, disinfectant, defoliant, anti-sapstain and anti-microbial agent. Pure PCP exists as colorless crystals with low solubility in water, but can be dissolved in organic solvents, such as alcohol, ether and benzene. It is also relatively volatile. Typically, commercial grade PCP is 86% pure [1,2].

In 1987, the annual consumption of PCP worldwide was estimated at 30,000 tons [1]. In the USA, PCP is no longer available for the general public and its use has been restricted [2]. Since 1991, the use of PCP in the EU has been severely restricted and its production has ceased. Countries such as Austria, Denmark, Finland, Germany, The Netherlands, Sweden and Switzerland have banned the use of PCP and PCP-containing products [3]. However, PCP is still used as a pesticide in some developing countries because of its low cost and various applications [4,5]. 

PCP as a wood and cellulose preservative was synthesized via two methods: (1) batch wise in nickel reactors by the chlorination of less halogenated CPs in the presence of aluminum trichloride or iron trichloride, known as the Boehringer process [6]; (2) through alkaline hydrolysis of hexachlorobenzene [1,7]. During the chlorination, some phenoxyphenols are formed, reacting further to produce PCDD/Fs [8]. The most abundant type of microcontamination during PCP synthesis is generally octachlorinated compounds [8] with concentrations up to 2500 mg/kg of octachlorinated dibenzo-*p*-dioxin (OCDD) [1]. Other abundantly detected dioxins are heptachlorodioxin (HeCDD), with concentrations up to 520 mg/kg, and even 2,3,7,8-tetrachlorodioxin (TeCDD) [1]. Chlorinated dibenzofurans are also formed; the most common are octachlorodibenzofurans (OCDF), with concentrations up to 260 mg/kg and heptachlorodibenzofurans (HeCDF), with concentrations up to 400 mg/kg [1]. These microcontaminants can impart a darker color to PCP crystals [2]. Generally, PCDD/Fs are present in low amounts; however, they are still a major concern due to their toxicity and persistence in the environment. In addition to PCP, other pesticides that are widely used may also contribute to the emission of PCDD/Fs. A study testing the presence of PCDD/Fs impurities in currently used pesticides found concentrations ranging from 0.020 to 2100 ng/g of active ingredient (AI, ΣPCDD/F) [9], with the highest concentrations found in pentachloronitrobenzene (PCNB). Yu et al. [8] discovered that the toxic equivalent (TEQ) concentrations become higher as the synthesis temperature increases. Therefore, it is recommended to keep the synthesis temperature as low as possible to minimize the production of PCDD/Fs during pesticide production.

Sawmills are facilities where wood is chopped and treated for preservation using pesticides; as mentioned above, PCP was first introduced as wood preservative and was broadly used worldwide. Therefore, it is common to find PCP and PCDD/Fs contamination near these facilities. Countries such as Finland and Sweden have had major contamination problems due to extensive use of wood preservatives containing PCP and its micropollutants. For instance, the main source of PCDD/Fs contamination in Finland has been the national production and use of Ky-5, a PCP-containing wood preservative during 1940–1984 [10]. In Sweden, this type of contamination is also a major problem, with approximately 400–500 wood impregnation sites [11]. A study performed near different sawmills in Scandinavia found concentrations of 500–3500 mg/kg d.w. of chlorinated phenols (CPs) and 0.2–5 mg/kg d.w. of polychlorinated dibenzofuran (PCDFs) in dry soil, concluding that the soil was heavily contaminated near these facilities [12]. In a more recent study of five contaminated sawmill sites in Sweden, the concentrations of CPs in the soil samples ranged from 0.1 to 4500 mg/kg d.w., PCDFs from 7.4 to 18,000 μg/kg d.w., and PCDDs from 9.9 to 35,000 μg/kg d.w. [11]. A study performed in the Kymijoki River in Finland showed heavy contamination mainly with Ky-5, a wood preservative agent containing higher chlorinated PCDD/Fs. The sediments from the river were contaminated mainly with 1,2,3,4,6,7,8-HeCDF, a major contaminant in the Ky-5 [13]. The mean total concentration at the most polluted river site downstream from the main source was 42,000 μg/kg d.w., indicating that this river is a major source of PCDD/Fs for the Gulf of Finland, and the concentrations found in the sediments are among the highest reported in the world [13]. Additionally, disposal of wood treated with PCP is still a large threat to the environment and human health. 

Unlike in Northern Europe where many sites are contaminated because of the wood industry’s activities, the main sources of PCP and PCDD/Fs contamination in Asia come from the production and use of pesticides. The total dioxin emissions from the use of pesticides in Japan during 1955–1995 was estimated at 540 tons of PCDD/Fs or 250 kg of TEQ from PCP [14]. A similar trend was found in the Yangtze River, where elevated concentrations of PCDD/Fs were found and one of the principal sources was the PCP production and use [15]. 

## 2. Toxicity and Bacterial Degradation of PCP

PCP can be found in all environments: soil, water, sediments and air; generally, environmental conditions are not favorable for its degradation. Factors such as microbial activity, PCP toxicity, terminal electron acceptors and soil properties influence the fate of PCP in the environment [16]. Complete mineralization of PCP and other CPs is possible through biodegradation, which is an effective and economic technique to remove these pollutants. PCP is more recalcitrant to bacterial degradation than less chlorinated CPs due to the presence of five chlorine atoms on the phenolic ring [17]. However, several strains of bacteria and fungi have been described in the literature as being able to degrade PCP aerobically or anaerobically. General toxicity of PCP is caused by its ability to act as an uncoupler of oxidative phosphorylation [18]. Low levels of dissolved oxygen, low pH and high temperature increase the toxic effects of PCP [1]. PCP is also bioaccumulated in aquatic organisms through the uptake from the surrounding water or along the food-chain [1]. Most aquatic invertebrates and vertebrates are affected by PCP concentrations below 1 mg/L in acute toxicity tests [1]. Fish species exhibit a 50% mortality rate at concentrations ranging from as low as 30 μg/L and as high as 600 μg/L [6,19]. In lower concentrations, PCP causes renal and hepatic lesions in fish [6]. Organisms in reproductive and juvenile stages are the most sensitive, with LC50 values as low as 0.01 mg/L for fish larvae [1]. Algal species appear to be the most sensitive aquatic organisms, as 1 μg/L can cause significant inhibition [1]. The EPA recommends that the maximum average PCP concentration in surface waters should not exceed 0.055 mg/L [6].

Inhalation exposures to PCP in humans have resulted in neurological, blood and liver defects, and eye irritation [2,20]. Long-term exposure to PCP by inhalation in humans has resulted in damage of the respiratory tract, blood, kidney, liver, immune system, eyes, nose and skin [2,20]. Studies conducted on humans have suggested that there is an association between exposure to PCP and cancer. For instance, research has demonstrated correlations between hematopoietic cancer and PCP exposure [21]. Chronic oral rodent studies have reported increases in liver tumors and two uncommon tumor types, adrenal gland pheochromocytomas in mice and mesotheliomas and nasal squamous cell carcinomas in rats [2,7,20].

### 2.1. Aerobic Bacterial Degradation of PCP 

**Figure 1:**
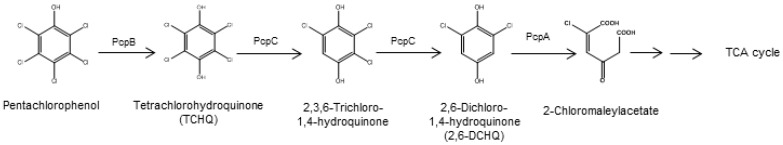
Bacterial degradation pathway of pentachlorophenol (PCP) in *Sphingobium chlorophenolicum* L-1. PcpB, PCP-4-monooxygenase; PcpC, tetrachlorohydroquinone (TCHQ) reductive dehalogenase; PcpA, 2,6-DCHQ 1,2-dioxygenase [17,23,29].

The first step of the biodegradation pathway of PCP in *Sphingobium chlorophenolicum* L-1, formerly known as *Sphingomonas chlorophenolicum* L-1 [22] (Figure 1), a NADPH-dependent conversion of PCP into tetrachlorohydroquinone (TCHQ) via the removal of chloride ions, is catalyzed by PCP 4-monooxygenase (PcpB) [23], a flavoprotein encoded by the *pcpB* gene [24,25,26]. Afterwards, TCHQ is sequentially dehalogenated into 2,6-dichloro-1,4-hydroquinone (2,6-DCHQ) by a TCHQ-reductive dehalogenase (RDase). This reaction is encoded by the *pcpC* gene [27]. In the next step, 2,6-DCHQ is degraded to 2-chloromaleylacetate by the 2,6-DCHQ-1,2 dioxygenase [28]. This step is crucial due to the cleavage of the aromatic ring. PcpE further converts 2-chloromaleylacetate into 3-oxoadipate via maleylacetate. Finally, 2-chloromaleylacetate is degraded in the tricarboxylic acid (TCA) cycle [17]. 

**Figure 2:**
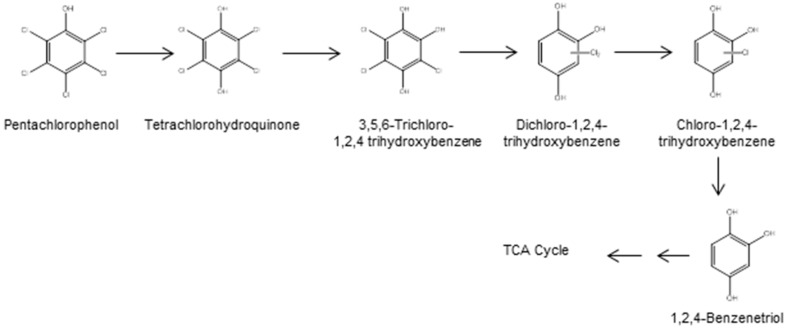
Bacterial degradation pathway of PCP in *Mycobacterium* strains [17,30,32].

There is a difference in the degradation pathway of PCP and the enzymes involved for Gram-positive bacteria. In *Mycobacterium chlorophenolicum* PCP-1 and *Mycobacterium fortuitum* CG-2, PCP is degraded by hydroxylation into TCHQ (Figure 2) by a membrane-bound cytochrome P-450 type enzyme [30,31]. In the next degradation step, TCHQ undergoes hydrolytic dehalogenation forming 3,5,6-trichloro-1,2,4-trihydroxybenzene [30,32], which is further transformed by reductive dehalogenation into dichloro-1,2,4-trihydroxybenzene [32]. Finally, after two successive reductive dehalogenation reactions, 1,2,4-benzenetriol is formed [32]. 

### 2.2. Anaerobic Reductive Dechlorination of PCP

CPs are often recalcitrant to aerobic bacterial attacks, but they can be reductively dehalogenated into phenols with less chlorine atoms, which may be further mineralized more easily [17]. 

Anaerobic conditions favor reductive dechlorination, resulting in the displacement of chlorine atoms by hydrogen atoms. Reductive dehalogenation involves the utilization of halogenated compounds as terminal electron acceptors for energy-conserving anaerobic respiratory electron transport [33]. Reductive dehalogenation can occur cometabolically, by bacteria triggering reductive dechlorination with unspecific enzyme systems, or catabolically, requiring the input of electron-donating substrates [34]. During reductive dehalogenation, electrons are transferred from an electron donor via an electron transfer chain to the membrane-localized RDase and, as a consequence, a proton gradient is generated across the membrane, which drives ATP formation through a membrane-bound ATPase [35,36,37]. This mechanism is dependent on the concentration and bioavailability of the organohalogen and can be stimulated by amendment with simple organic growth substrates [38]. Organohalide respiring bacteria cluster with the genera *Desulfitobacterium*, *Dehalobacter*, *Anaeromyxobacter*, *Geobacter*, *Desulfomonile*, *Desulfuromonas*, *Desulfovibrio*, *Sulfurospirillum*, *Dehalogenimonas*, *Dehalobium* and *Dehalococcoides* [39]. 

**Figure 3:**
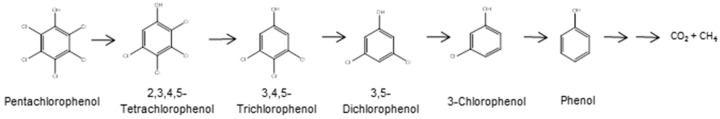
Anaerobic dehalogenation of PCP [17,40].

PCP is anaerobically degraded through a series of reductive dehalogenation reactions, resulting in the formation of phenol, which can further be degraded to CH_4_ and CO_2_ by anaerobic prokaryotes (Figure 3) [40,41,42]. Several bacteria with the capacity for dehalogenation of PCP have been isolated, from which those of the *Desulfitobacterium* genus have been widely studied. *Desulfitobacterium hafniense* strain PCP-1 showed two different enzymatic systems involved in PCP dechlorination, one dechlorinates PCP at the *ortho* position to generate 3,4,5-TCP and the second dechlorinates 3,4,5-TCP at the *meta* and *para* positions to generate 3-CP. PCP, 2,4,6-TCP, 2,3,4-TCP, 2,3,5-TCP, 2,6-DCP and 2,4-DCP were found to be the inducers of the *ortho*-dechlorinating activity, while only 3,4,5-TCP and 3,5-DCP were reported to induce *meta*- and *para*-dechlorinating activities [42]. 

Reductive dehalogenation of chloroaromatic and chloroalkyl compounds is usually carried out by RDases. One of these RDases is *ortho*-CP RDase, which is the terminal reductase involved in the halorespiratory chain of *Desulfitobacterium dehalogenans* and mediates the electron transfer from an electron donor to a halogenated substrate [43]. The genes clustering with this RDase are c*prA*, encoding the *o*-CP RDase and *cprB*, coding for an integral membrane protein that can act as a membrane anchor of the dehalogenase [43]. Four gene loci encoding putative CP-RDases (CprA 2–5) were screened in *D. hafniense* strain PCP-1. CprA3 is involved in the dechlorination of highly chlorinated phenols with high affinity toward PCP [44], while CprA5 catalyzes the dechlorination of CPs at the *meta* and *para* positions [45]. For CprA2 and CprA4, there is yet to be any evidence of dehalogenation; however, phylogenetic analyses have grouped them with the *ortho*-dechlorinating CprA, suggesting similar activities to those of CprA3 and CprA5 [42,44].

Boyer et al. [46] isolated another RDase, named CrdA, from *D. hafniense* strain PCP-1 cultures induced for *ortho*-dechlorinating activities with 2,4,6-TCP. This RDase did not show any homology with any known dehalogenase, suggesting distinct RDases, which dechlorinate PCP and 2,4,6-TCP at the *ortho* positions [42]. 

## 3. Biodegradation of Polychlorinated Dibenzo-*p*-Dioxins (PCDDs) and Polychlorinated Dibenzofurans (PCDFs)

**Figure 4:**
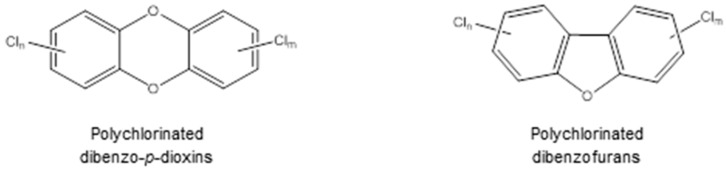
Chemical structure of PCDD/Fs.

PCDD/Fs (Figure 4) are organohalogenated substances, which form a group of 210 different congeners: 135 belong to PCDFs and 75 to PCDDs, each differing in the number and position of the chlorine atoms. In PCDDs, the benzene rings are connected by two oxygen bridges and, in PCDFs, the benzene rings are linked by a carbon bond and an oxygen bridge. 

PCDD/Fs are colorless organic solids with a high melting point, low vapor pressure, and very low water solubility. PCDD/Fs enter the environment as mixtures containing a variety of individual components and impurities [47]. Due to their properties, PCDD/Fs are toxic and persistent in the environment with prone absorbability to particulate matter, soil particles and other surfaces. The highly chlorinated dioxins and furans are more strongly bound onto fractions of amorphous organic carbon and black carbon in soils and sediments. Consequently, their bioavailability and biodegradability are reduced [48,49]. The water solubility of PCDD/Fs decreases and their solubility in organic solvents and fats increases as the chlorine content grows [50,51]. The main origin of PCDD/Fs is from unintentional by-products of industrial processes and incomplete combustion, but they also have natural sources, such as forest fires and volcano eruptions [52]. As previously stated, PCDD/Fs are also found as impurities in pesticides, such as PCP. Consequently, PCDD/Fs primarily accumulate on the surface soil nearby wood preserving facilities as a result of impregnation activities [12]. Another source of PCDD/F emission related to PCP comes from wood combustion, accidental fires, and from the combustion and landfilling of PCP-treated wood; however, these emissions are difficult to assess [53]. The PCDD/Fs congener profiles do not match any known natural or anthropogenic source and global PCDD/Fs budgets fail to account for the observed levels in soils. Therefore, there is the hypothesis that clay minerals play a central role in the natural in situ synthesis of PCDD/Fs [54,55]. Recently it was also found that PCDD/Fs, especially OCDD, were formed when PCP was mixed with clay minerals (Fe(III)-montmorillonite) [54]. This reaction is initiated by single electron transfer from PCP to Fe(III)-montmorillonite, thereby forming the PCP radical cation. Subsequent dimerization, dechlorination, and ring closure reactions result in the formation of OCDD [54]. In another study, Gu et al. [55] demonstrated the formation of precursors of TeCDD and PeCDD when 2,4,5-TCP was mixed with a smectite clay under environmental conditions. These findings support the hypothesis of in situ PCDD formation in soils and geologic clay deposits [54,55]. 

### 3.1. PCDD/Fs Toxicity

Dioxins are persistent, toxic, bio-accumulative, can volatilize and be transported over long distances from the source of emission [53]. In humans and other vertebrates, PCDD/Fs showed to be a risk factor for cancer, immune deficiency, central and peripheral nervous system pathology, endocrine disruption, decreased pulmonary functions and bronchitis, altered serum testosterone levels, eyelid pathology, gum pigmentation, skin rashes, hypertrichosis, liver damage, adverse cardiovascular effects and elevated serum cholesterol and triglycerides [47,50,56,57]. Seventeen of the isomers showed to be extremely toxic, mutagenic and linked to the suppression of the human immune system [53,57]. Other effects address impairment of the developing nervous system, the endocrine system and reproductive functions [57]. In the environment, PCDD/Fs tend to bind to organic matter in sediments and soils and to accumulate in fatty tissues of living organisms. The toxic responses of some animals include: body weight loss, hepatotoxicity, porphyria, dermal toxicity, gastric lesions, thymus atrophy and immunotoxicity, teratogenicity, reproductive effects, and carcinogenicity [58]. 

Dahlgren et al. [59] conducted an exposure assessment in residents living near a wood treatment plant and found elevated levels of highly chlorinated dioxins, especially HeCDD and OCDD in some individuals, documenting a substantial contamination of the neighborhood of wood treatment plants by wood processing waste chemicals. Cattle have been found with elevated levels of PCDD/Fs concentrations, probably coming from PCP-treated wood used in stables, which represents another source of contamination for humans [60,61].

### 3.2. Biodegradation of PCDD/Fs

Under aerobic conditions, less chlorinated dioxins are susceptible to partial degradation during cometabolic metabolism, while highly chlorinated dioxins tend to be more easily degraded by reductive dehalogenation. In a few cases, PCDD/Fs have been reported to serve as growth substrates, and these cases are restricted to mono-, di- and tri-chlorinated congeners [33,56,62,63,64]. 

#### 3.2.1. Aerobic Bacterial Degradation of PCDD/Fs

Oxidative degradation of DD and DF occurs in two major ways. One is termed lateral dioxygenation whereby one of the aromatic rings is attacked at the lateral 1,2 or 2,3 or 3,4 positions. The second way is via angular dioxygenation, where initial hydroxylation starts at the angular 4 and 4a positions adjacent to the ether bridge [65]. Lateral dioxygenation has been reported in several bacterial strains to produce a yellow metabolite [65,66]. 

**Figure 5:**
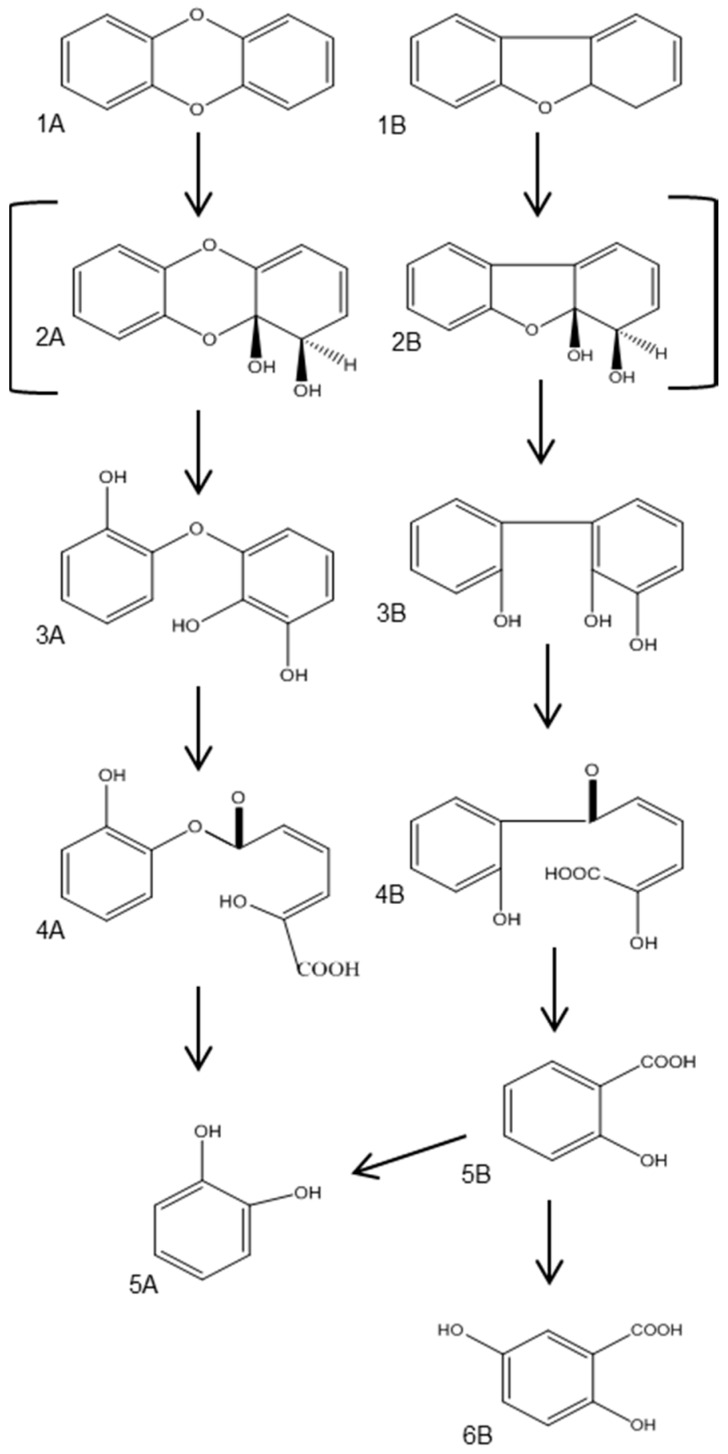
Degradation pathway of dibenzo-*p*-dioxin (**1A**); and dibenzofuran (**1B**) [56,62,65,66,68,69,70]. Compound definition: cis-dihydrodiols (**2A**,**2B**); 2,2’,3-trihydroxydiphenyl ether (**3A**); 2,2’,3-trihydroxybiphenyl (**3B**); 2-hydroxy-6-oxo-6-(2-hydroxyphenoxy)-hexa-2,4-dienoic acid (**4A**); 2-hydroxy-6-oxo-6-(2-hydroxyphenyl)-hexa-2,4-dienoic acid (**4B**); catechol (**5A**); salicylic acid (**5B**); gentisic acid (**6B**).

The biodegradation pathways of aerobic angular dioxygenation of dioxins (Figure 5, 1A) and furans (Figure 5, 1B) are illustrated in Figure 5. Klečka and Gibson [67] reported the co-oxidation of DD to cis-1,2-dihydroxy-1,2-dihydrodibenzo-*p*-dioxin and 1,2-dihydroxydibenzo-*p*-dioxin by a *Pseudomonas* species. The dihydrodiols formed are also products in the oxidation and co-oxidation of dibenzofurans and other aromatic compounds, such as naphthalene and biphenyl [56,65,68,69]. Rieske non-heme iron oxygenases are responsible for catalyzing the first reaction in the degradation pathway of DD/Fs [62,65,69]. This step is crucial in the degradation pathway because it destroys the planar structure of the dioxins and furans, which makes these compounds toxic [69]. The next step is catalyzed spontaneously and produces 2,2’,3-trihydroxydiphenyl ether (Figure 5, 3A) from DD and 2,2’,3-trihydroxybiphenyl (Figure 5, 3B) from DF [68]. In the next step, dioxygenases cause the ring opening, producing 2-hydroxy-6-oxo-6-(2-hydroxyphenoxy)-hexa-2,4-dienoic acid (Figure 5, 4A) and 2-hydroxy-6-oxo-6-(2-hydroxyphenyl)-hexa-2,4-dienoic acid (Figure 5, 4B) in the case of DD and DF, respectively [56,62,65,68]. These compounds are further degraded to catechol (Figure 5, 5A) and salicylate (Figure 5, 5B) by hydrolysis [56,65,68]. Salicylate is then degraded via the gentisate pathway or converted to catechol. Chlorinated dioxins rarely serve as a sole source of carbon and energy; however, Hong et al. [63] found that *Pseudomonas veronii* PH-03 can utilize 1-CDD and 2-CDD. 

**Figure 6:**
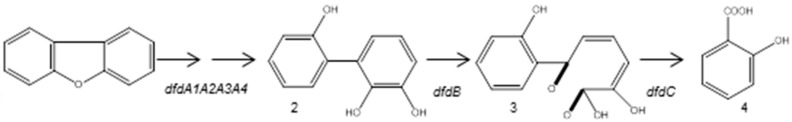
Proposed degradation pathway of DF by *Nocardioides* sp. DF412. Genes involved in each reaction are specified below each arrow. Compound definition: dibenzofuran (**1**); 2,2’,3-trihydroxybiphenyl (**2**); 2-hydroxy-6-(2-hydroxyphenyl)-6-oxo-2,4-hexadienoic acid (**3**); and salicylate (**4**) [71].

An alternative degradation pathway for DF was reported in *Nocardioides* sp. strain DF412 [71,72]. This strain is able to grow on DF and salicylate, and has two gene clusters involved in the degradation of DF. The gene cluster *dfdA1A2A3A4* codes for ring hydroxylating dioxygenase components, whereas the *dfdBC* cluster codes for the ring-cleavage dioxygenase and hydrolase, respectively [71]. The proposed DF degradation pathway is depicted in Figure 6 [71], and the same pathway was found operating in *Serratia marcescens* [73]. 

The genes encoding dioxygenases in the degradation pathway of DD and DF have also been studied in *Sphingomonas wittichii* RW1, formerly called *Sphingomonas* sp. strain RW1 [74], and several *Terrabacter* spp. A study by Armengaud et al. [75] showed that genes encoding the electron supply system of the dioxygenase are not clustered with the dioxygenase gene, but are located on two other separate genome segments, which contrasts with the typical genetic organization of catabolic pathways, where genes tend to be clustered. Furthermore, Armengaud et al. [76] studied *dxnA1A2* locus, which encodes the dioxygenase components of the initial dioxygenase system of DD and DF catabolic pathways. The study showed that this fragment contains ten collinear open reading frames (ORFs), apparently organized in one compact operon; *dxnB*, *dxnC-dxnI*, ORF2, and *fdx3.* In *Terrabacter* sp. strain DBF63, *dbfA1A2* encodes the oxygenase component of a multicomponent dioxygenase, *dbfBC* encodes for a *meta* cleavage enzyme and hydrolase and *pht* genes encode for phthalate-degrading enzymes. Proteins DbfA1A2 and DbfBC are responsible for catalyzing the conversion of DF to salicylate, while DbfA1A2 and Pht enzymes are involved in fluorene degradation [69]. *dbfA1A2* cistron and *pht* operon are located on the two linear plasmids, while *dbfBC* genes are located on the chromosome of *Terrabacter* sp. strain DBF63 [69]. 

Aerobic biodegradability of PCDD/Fs, which occurs via cometabolism, increases with a decreasing number of chlorine atoms, leaving PCDD/Fs with five chlorine atoms or more not prone to aerobic degradation [62,77]. However, there are a few exceptions, such as the study performed by Nam et al. [78], which demonstrated that *Sphingomonas wittichii* RW1 is able to aerobically catabolize 1,2,3,4,7,8-HxCDD. The most widely used primary substrate for the cometabolism of PCDD/Fs has been non-halogenated DF [62]. 

#### 3.2.2. Anaerobic Reductive Dechlorination of PCDD/Fs

The most promising approach to biodegradation of highly chlorinated dioxins is reductive dehalogenation using anaerobic bacteria [79]. PCDD/Fs, similarly to PCP, can be degraded by stepwise reductive removal of halogen atoms. PCDD/Fs, like other halogenated compounds, can serve as terminal electron acceptors in dehalorespiration [35,36].

**Figure 7:**
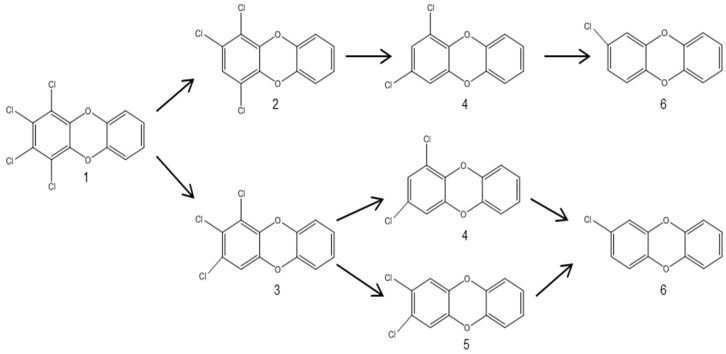
Proposed pathway for the dechlorination of 1,2,3,4-TeCDD (**1**) and 1,2,3-TCDD (**3**) by the anaerobic consortium of river sediment from the Saale River [79,81,82]. Compound definition: 1,2,3,4-tetrachlorodibenzo-*p*-dioxin (1,2,3,4-TeCDD) (**1**); 1,2,4-trichlorodibenzo-*p*-dioxin (1,2,3-TCDD) (**2**); 1,2,3-trichlorodibenzo-*p*-dioxin (1,2,3-TCDD) (**3**); 1,3-dichlorodibenzo-*p*-dioxin (1,3-DCDD) (**4**); 2,3-dichlorodibenzo-*p*-dioxin (2,3-DCDD) (**5**); 2-monochlorodibenzo-*p*-dioxin (**6**).

Dechlorination and the order of chlorine removal from 1,2,3,4-TeCDD (Figure 7, 1), 1,2,4-TCDD (Figure 7, 2), and 1,2,3-TCDD (Figure 7, 3) was studied by Ballerstedt et al. [79] using enrichment cultures from sediment of the Saale River. The study showed that dechlorination by this culture proceeded via both 1,2,4-TCDD (Figure 7, 2) and 1,2,3-TCDD (Figure 7, 3). Reductive dechlorination of 1,2,4-TCDD yielded 1,3-DCDD, while 1,2,3-TCDD was dechlorinated to 1,3-DCDD (Figure 7, 4) and 2,3-DCDD (Figure 7, 5). These results suggested that the main dechlorination route of 1,2,3,4-TeCDD to 1,3-DCDD proceeded primarily via the removal of a lateral chlorine atom. The same intermediates were found in a microcosm experiment with sediments from the River Kymijoki [80], and similar results were obtained in a study by Beurskens et al. [81] with anaerobic microorganisms enriched from the Rhine River sediments; however, dechlorination occurred simultaneously in lateral and *peri*-positions, which is the closest to the ether bridge. The same pathway of simultaneous dechlorination in lateral and *peri*-positions was found by Bunge et al. [82]; however, it was accompanied by the degradation of 1,2,3,4-TeCDD into 1,2,4-TCDD, which accumulated and was not dehalogenated any further. 

**Figure 8:**
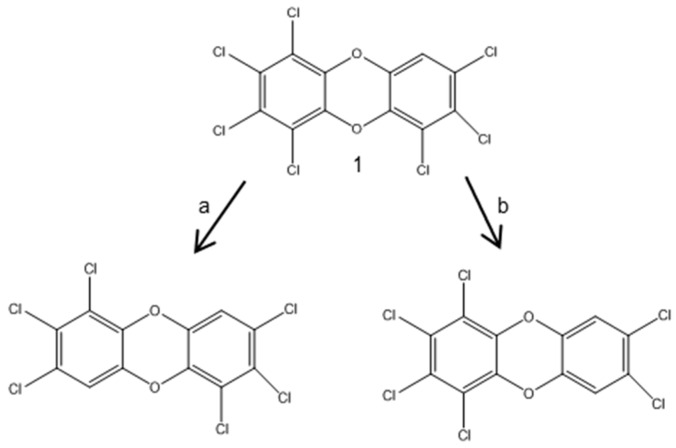
Proposed dechlorination of 1,2,3,4,6,7,8-HeCDF (**1**) from aquifer microcosms [38]. The major dechlorination pathway is (**a**); and the minor is (**b**).

Different dechlorination pathways found in one sediment core suggested that different dechlorinating populations were involved in these processes. It was also found that the position and congener specificity of microbial PCDD/Fs’ reductive dechlorination might depend on specific bacteria [82]. Further support to this theory was given in a study performed on higher chlorinated dioxins (Figure 8), which showed that *peri*-dechlorination was the preferential route of reduction in 1,2,3,4,6,7,8-HeCDF [38]. Such diversity of dechlorination pathways demonstrates a large microbial degradation potential with preferences for particular substitution patterns under anaerobic conditions, indicating different strategies of bacterial adaptation to natural or anthropogenic organohalogen compounds [36].

Catabolism of persistent chlorinated aromatics and aliphatics by reductive dechlorination was widely studied in the genus *Dehalococcoides*. Organohalide respiration in *Dehalococcoides* is catalyzed by heterodimeric, membrane-bound RDases of about 500 amino acids in length [83,84]. It is believed that all RDases have a catalytically active “A” subunit anchored to the outside of the cytoplasmic membrane by a predicted integral membrane “B” subunit [37,85]. RDases from *Dehalococcoides*, *Dehalobacter*, and *Desulfitobacterium* strains are among the most studied ones [37]. The genome of *Dehalococcoides mccartyi*, formerly known as the *Dehalococcoides ethenogenes* strain 195 [86], contains genes encoding 17 putative RDases [87], while *Dehalococcoides* sp. strain CBDB1, which dechlorinates dioxins, was found with 32 *rdh* genes [88]. Comparative analysis of *Dehalococcoides mccartyi* and *Dehalococcoides* sp. strain CBDB1 genomes revealed a high degree of gene context conservation as well as high plasticity in all regions containing *rdh* genes, suggesting that these regions are under intense evolutionary pressure [88]. *Dehalococcoides* strain VS, in contrast, was found with 36 full-length *rdhAB* [83]. The presence of between 11 and 36 *rdhAB* per genome implies a respiratory flexibility that allows *Dehalococcoides* to use a variety of halogenated compounds as terminal electron acceptors [83]. 

*Dehalococcoides* and *Dehalobacter* are strictly dependent on reductive dechlorination. Studies on their genomes suggest that they are also restricted to H_2_ as their electron donor [39]. There is still very little information on the substrate specificity of respective RDases. A recent study [37] performed a maximum likelihood-based phylogenetic analysis of amino acid sequences of RDases. The results showed two main clusters, one with specificity to substrates such as chlorinated methanes, ethanes, propanes, ethenes, chlorinated benzenes and meta/para-chlorophenols. Surprisingly, all RDases studied in *Dehalococcoides* were included in this cluster, forming a separate clade. The second cluster contained all CprAses and RDases identified in aerobic microbes. To the best of our knowledge, there is no information on the specific RDases dechlorinating PCDD/Fs. However, this is not surprising as just in 2014 there was a study by Wang et al. [89] which identified and confirmed novel genes encoding RDases for catalyzing PCB dechlorination.

## 4. Bioremediation Studies

As mentioned previously, PCP is a pesticide used as a wood preservation agent, which forms PCDD/Fs as micropollutants during its production. In consequence, it is common that in close proximity to wood preserving facilities, PCP and PCDD/Fs have accumulated in the soil as result of impregnation. Several bioremediation techniques have been tested with contaminated soil from former sawmills, as well as soil in close proximity to PCP-production facilities or soil and sediments intentionally spiked with PCP or PCDD/Fs. Some of these techniques include: composting, soil biopiles, bioaugmentation, biostimulation, phytoremediation and use of bioreactors, with specific examples given below. 

### 4.1. Composting and Soil Biopiles

Laine and Jørgensen [90] studied the use of straw compost and remediated soil as inoculums to bioremediate CP-contaminated soil under field-simulating conditions. PCP-adapted straw compost mineralized 56% of the added PCP in 4 weeks and no partial dechlorination products of PCP were found. In a further study by Laine and Jørgensen [91], a pilot-scale composting system of CP-contaminated soil was performed. The objective was to compare CP degradation when indigenous soil microbes from two different inoculants (straw compost and remediated soil) were used. It was found that over 90% of the CPs were removed during the composting period, regardless of the inoculum. Jaspers et al. [92] also reported an important decrease of PCP during composting with farmyard manure, followed by the appearance of tetra and triCPs. These results are similar to those found by Zeng et al. [93] with PCP removal of around 72% and the highest removal rate occurring at the thermophilic phase of composting. In another investigation of the fate of CPs during composting of sawmill soil and impregnated wood, Laine et al. [94] studied whether CPs form any harmful metabolites. During composting of CP-contaminated soil no harmful metabolites were formed. PCDD/Fs were also analyzed in the compost piles and it was found that no degradation occurred during this process. In a more recent study, Narihiro et al. [95] studied a semi-aerobic, mesophilic, fed-batch composting (FBC) reactor to bioremediate heavily PCDD/F-contaminated solid materials. The initial concentrations of PCDD/Fs were 200–800 pmol/g d.w. and after a year of operation were reduced to 15%–16% with a half-reduction time of 4 months. The reactors produced only small amounts of mono- to tri-CDD/Fs, suggesting a complete mineralization of PCDD/Fs due to a combination of reductive dechlorination of polychlorinated congeners and oxidative degradation of the dechlorinated products. 

A similar strategy to composting is soil biopiles; controlled engineered bioprocesses in which biodegradable contaminants can be mineralized by microorganisms under aerobic conditions. Miller et al. [96] performed a study that tested the effect of selected nutrient amendments and temperature on the biodegradation of PCP in contaminated soil within a soil biopile on a laboratory scale. The different nutrient amendments tested were: (1) control (no additions); (2) inorganic amendment; (3) aged dairy manure amendment; and (4) Municipal Solid Waste (MSW) compost amendment. The temperatures tested were 10, 15, and 20 ± 1 °C. It was demonstrated that the temperature did not have a significant effect on the removal of PCP; however, the nutrient amendments did have a significant effect. The best result was from the MSW compost, in which PCP decreased by 76% in the amended soil, while the concentration of inorganic chloride ions increased. 

Another approach was tested by Sinkkonen et al. [97] who investigated whether the activity of microorganisms from organic soil with high degradation capacity would enhance degradation of recalcitrant CPs in the contaminated soil. They found that after a thin layer of the pine forest humus was added on the top of mineral sawmill soil, original CP concentrations (70–20 μg/g) decreased by >40% in 24 days. No degradation was found in this soil, when either it was kept bare or covered with the pine forest humus soil layer previously sterilized.

### 4.2. Bioreactors and Microcosms

Kao and Wu [98] developed a method that combines partial oxidation using Fenton’s Reagent and bioremediation to efficiently remediate TeCDD-contaminated soils in bioreactors. They observed that up to 99% of TeCDD was transformed to less-chlorinated and less-toxic byproducts (chlorobenzenes and CPs) after chemical pre-treatment, promoting their bioavailability to microbial communities. During the biodegradation phase, the transformation of chlorinated compounds was observed after a few days of incubation in the bioreactors. The authors concluded that the two-stage partial oxidation followed by biodegradation has the potential to be developed as a technique to remediate TeCDD-contaminated soils on-site. A further study by Kao et al. [99] evaluated the effectiveness of using bioremediation to clean up TeCDD-contaminated soils under different reduction/oxidation conditions and different inoculums, such as activated sludge and aquifer sediments from a TeCDD- and a PCP-contaminated site or by using different primary substrates to enhance the reductive dechlorination of TeCDD, such as acetate, sludge cake, and cane molasses. The highest TeCDD biodegradation efficiency was observed in microcosms containing activated sludge as inoculum and sludge cake as carbon source.

In a study by Chen et al. [100], biodegradation was tested on soil heavily contaminated with PCDD/Fs, due to a historically large production of PCP. Most of the forms of PCDD/Fs in the soil were OCDD, OCDF and HeCDF. Indigenous microbial activities in the soil microcosm experiment were stimulated by the addition of oxygen and nutrients (first batch microcosms). Results demonstrated that after 6 weeks, almost 100% of OCDF and 97.75% of HeCDFs were reduced, but no degradation of OCDD occurred within 12 weeks. During degradation of highly chlorinated PCDD/Fs, concentrations of less chlorinated congeners of DD/Fs increased. In a further study that aimed to validate the degradation of OCDF, a previous inoculum was added to soil in addition to OCDF. OCDF was degraded with an efficiency of 82.1% and OCDD concentration decreased by 87.9%. That inoculum was likely able to degrade OCDD due to a previous acclimation procedure with those PCDD/Fs which are more easily degraded and have lower initial concentrations of OCDD. Toxicity decreased in this study in comparison with the first batch microcosms. In another study by Chen et al. [101] compost-amended landfill reactors were constructed to degrade PCDD/Fs in contaminated soil. The first part of the study attempted to choose the best compost that could be used later in the reactors based on the removal of PCDD/Fs. The best removal efficiency (~61%) was found in waste sludge (WS) from a pulp and paper wastewater treatment plant compost followed by plant leaf (PL) and cattle manure (CM) with removals of 36% and 32%, respectively, after 30 days. Based on these results, WS and CM composts were chosen. The second part of the study was focused on the efficiency of three compost-amended landfill reactors to remove PCDD/Fs. The reactors containing soil were named RS (no amendment), RSW (with waste sludge compost) and RSWC (with waste sludge and cattle manure compost). The conditions in the reactors were hypoxic by maintaining a periodic leachate circulation and aeration. After 280 days the removal was 33.3% for RS, 70.5% for RSW and 78.9% for RSWC.

Binh et al. [102] investigated the biodegradation of dioxins in contaminated soil under sequential anaerobic (17 weeks) and aerobic conditions (6 weeks). For this purpose, microcosms under different treatments were studied as follows: soil spiked with 2,3,7,8-TeCDD and nutrients; spiked soil plus carboxymethylcellulose (CMC) coated on nanoscale zero valent iron (nZVI); spiked soil plus Tween-80 (surfactant); and spiked soil plus CMC-coated nZVI and Tween-80. All treatments proved efficient in removing 2,3,7,8-TeCDD after the sequential anaerobic and aerobic treatment periods with a removal ranging between 40% and 60%. The study concluded that the enrichment with microorganisms collected from the dioxin-contaminated site were effective for the removal of dioxins.

Ahn et al. [80] performed a study to evaluate the dechlorination of 1,2,3,4-TeCDD-spiked historically PCDD/F-contaminated sediments undergoing stimulation through the addition of alternate halogenated electron acceptors and/or bioaugmentation with *Dehalococcoides mccartyi*. Sediments from the River Kymijoki had the highest dechlorination removal of 1,2,3,4-TeCDD, reaching 76.4%–88.2% removal after 120 days. This study provided evidence of a combined bioaugmentation/biostimulation approach for the bioremediation of contaminated sediment with PCDD/Fs. The authors also found that the addition of halogenated co-amendments might be a tool to enhance dechlorination of PCDD/Fs by promoting the growth of dehalogenating bacteria. Liu et al. [103] assessed the dechlorination of 1,2,3,4-TeCDD/F and OCDF by enrichment cultures derived also from the River Kymijoki sediment. The purpose of the work was to examine the effect of pentachloronitrobenzene (PCNB) on the dechlorination and how the microbial community responded to different treatments. The main results demonstrated the occurrence of both microbial reductive dechlorination of 1,2,3,4-TeCDF, 1,2,3,4-TeCDD and OCDF, and the stimulating effect of PCNB in the enrichment cultures from the River Kymijoki.

### 4.3. Phytoremediation

Phytoremediation has been frequently studied for its potential to bioremediate soil by aromatic compounds [104,105,106,107,108]. In a study by Wang and Oyaizu [109], the phytoremediation potential of four plant species was evaluated in a DF-spiked soil. The study showed that white clover (*Trifolium repens* L.) outcompeted the other plants—bermuda grass (*Cynodon dactylon*), bent grass (*Agrostis palustris* Huds.), and lawn grass (*Zoysia japonica*)—in terms of root biomass and numbers of DF-degrading bacteria. As a result, planted non-sterile soil showed a significant reduction in DF compared to the unplanted non-sterile soil. Furthermore, white clover-planted contaminated soil exhibited a high rate of DF removal (66% after 60 days). It was also found that microbial populations capable of degrading DF were selectively enriched by the addition of DF. In a subsequent study by Wang and Oyaizu [110], the degradation ability of *Comamonas* sp. strain KD7, which was hypothesized to form biofilms on the surface of white clover roots, was examined. The aim was to provide experimental evidence for improved rhizoremediation and to develop an alternative approach to remediate PCDD/F-contaminated soils using a plant-microbe system. For that purpose, soil was spiked with 2,8-DCDF, 1-CDD, 2,7-DCDD, 1,2,4-TCDD, and 1,2,3,4-TeCDD with initial concentrations of 20 mg/kg, and DF with a concentration of 1000 mg/kg, and four treatments were tested: (1) unplanted and uninoculated; (2) unplanted and strain KD7 inoculated; (3) white clover planted and uninoculated; and (4) white clover and strain KD7 inoculated. The results revealed that the white clover seeds inoculated with strain KD7 exhibited higher germination efficiency and increased root elongation compared with uninoculated white clover. Regarding the removal of contaminants, a significant decrease of DF was observed in unplanted soil inoculated with strain KD7 (63%) compared to unplanted and uninoculated soil. The variant of white clover-planted soil inoculated with strain KD7 resulted in significant reductions in DF (43%), 2,8-DCDF (68%) and DD (58%).

## 5. Microbial Communities in Contaminated Environments

The study of microbial communities in contaminated environments with PCDD/Fs is important to observe the phylogeny of possible biodegraders. The following studies have provided insight into the composition and structure of bacterial communities found in soils contaminated with PCDD/Fs.

### 5.1. Bacterial Communities In Situ

Futamata et al. [111] performed a study on the distribution of DF-degrading bacteria in PCDD/F-polluted soils in Japan. Concentrations of PCDD/Fs in the soils ranged from 6.8 to 4600 pg TEQ/g d.w. The results demonstrated that 78% of the isolates clustered with members of the class *Actinobacteria*, mainly the genera *Nocardioides* and *Rhodococcus*. Other isolates belonged to the genera *Arthrobacter* and *Janibacter*, with the rest being identified as members of the family *Sphingomonadaceae*. Notably, a higher number of DF-degrading bacterial isolates were separated from the most polluted samples. A similar study was carried out by Hanano et al. [112] with sampling sites located close to potential industrial sources of PCDD/Fs in Syria. The results of dioxin concentrations varied from 2.5 to 50 ppt and the highest concentrations were found near the vicinity of Homs refinery. The bacterial community in all sites was dominated mainly by *Bacillus* genus (74.7%), followed by the genera *Arthrobacter* and *Klebsiella*—5.2% and 4.7%, respectively. *Bacillus subtilis*, *Bacillus simplex*, *Bacillus thuringiensis*, *Bacillus megaterium*, *Arthrobacter aurescens*, *Klebsiella oxytoca*, *Pantoea calida*, *Enterobacter cloacae* and *Microbacterium foliorum* were the most abundant species. It was found that the majority of bacterial species containing angular dioxygenase encoding gene were detected in the most polluted soils.

### 5.2. Bacterial Communities in Bioremediation Studies

In a further study to understand the microbial community structure of the previously mentioned microcosm degrading OCDF, Chen et al. [113] pyrosequenced 16S rRNA genes of the bacterial communities transited from polluted soil to the batch microcosms. Results revealed that although oxygen was provided, anaerobic *Sedimentibacter* initially emerged as the abundant pioneer. Aerobic genera exhibited an increase in their 16S rRNA gene copies within the timeframe of the study, which indicates their involvement in the degradation of OCDF under hypoxic conditions. Chen et al. [101] also studied the microbial community in the compost-amended landfill reactors to degrade PCDD/Fs in contaminated soil. The predominant phyla found in the three reactors initially consisted of *Proteobacteria*, *Firmicutes*, and *Actinobacteria*. The latter remained dominant until the end of the operation but experienced remarkable changes in terms of abundance. The inoculation with waste sludge (WS) and cattle manure (CM) compost made bacilli-related population abundant. *Gammaproteobacteria*- and *Chloroflexi*-related populations were enriched in reactor RSW (with waste sludge compost) while *Alphaproteobacteria*-related population was the one enhanced in reactor RSWC (with waste sludge and cattle manure compost). There were high sequence frequencies of potential degraders such as *Bacillus*, *Pseudomonas* and *Chloroflexi* phylum.

Yoshida et al. [114] constructed anaerobic microcosms containing river sediments heavily polluted with PCDD/Fs. The aim was to study the dechlorination of these compounds and the microbial community structure. The reduction of PCDD/Fs was about 49% of the initial concentration after 360 days without the accumulation of less-chlorinated congeners as intermediates or end products. The half-reduction time was estimated to be 14 months. Major phyla found were *Firmicutes*, *Proteobacteria* and *Bacteroidetes*; however, *Chloroflexi* and *Dehalococcoides* were also found in the microbial community. This suggests that anaerobic bacteria with the capacity of reductively dechlorinate PCDD/Fs and aerobic bacteria with the oxidation capacity towards PCDD/Fs probably coexist and can completely mineralize these compounds. Another study using semi-anaerobic microcosms from lake sediments under different levels of PCDD/F contamination [115] aimed to foresee kinetic patterns of PCDD/F degradation in the microcosms and to study the relationship between the level of contamination and the density of *Dehalococcoides*. PCDD/Fs had similar reducing coefficients with only trace amounts of less chlorinated congeners as intermediates or end products. Major phyla in the bacterial community were *Bacteroidetes*, *Firmicutes* and *Proteobacteria* regardless of the PCDD/Fs concentrations. The population density of *Dehalococcoides* was correlated with PCDD/F concentration. The study concluded that under semi-anaerobic conditions, anaerobic reductive dechlorinating bacteria and aerobic bacteria with the oxidation capacity towards PCDD/Fs could develop and apparently completely mineralize PCDD/Fs. In a more recent experiment by Kaiya et al. [116], aromatic-hydrocarbon-degrading (AHD) bacteria from semi-anaerobic PCDD/F-transforming microcosms were studied. The microcosms were inoculated with river sediment and incubated for 1.6 years with dibenzofuran as the model substrate. During this period the concentration of PCDD/Fs in the microcosm decreased to 43% on a molar basis and no significant accumulation of mono- to tri-CDD/Fs as intermediate dechlorinated products were detected. DF-degrading isolates were identified as members of the phyla *Actinobacteria*, *Firmicutes* and *Proteobacteria*, with the genera *Paenibacillus* and *Rhizobium* being the most abundant isolates. 

Microbial populations were also analyzed in the study, operating semi-aerobic FBC reactors to bioremediate heavily PCDD/F-contaminated solid materials [95]. The major populations found in the FBC reactors shifted from the *Proteobacteria* to *Actinobacteria* and, during the overall period, *Bacteroidetes* and *Firmicutes* phyla were present, with possible dechlorinators being *Dehalococcoides* and *Chloroflexi*.

## 6. Conclusions

Soils close to sawmills tend to be heavily contaminated with PCP, other CPs and PCDD/Fs. However, there appears to be a diverse spectrum of microorganisms capable of degrading these pollutants, indicating that bioremediation of such contaminated localities is possible even by natural attenuation. In order to increase the effectiveness and degradation rate, several studies have tested different technologies and conditions. Engineered biosystems have also attracted attention for their potential application in bioremediation of PCDD/Fs. In this review we are providing an overview of different bioremediation approaches with a couple of acknowledged conclusions, such as the complete mineralization of PCP and PCDD/Fs through a combination of reductive dechlorination of polychlorinated congeners and oxidative degradation of the dechlorinated products. Mineralization can also be achieved by amending contaminated soil with organic soil rich in nutrients and high in microbial diversity.

Summarizations have been made suggesting that links exist between successful removal of PCP or PCDD/Fs and specific microbial populations; however, further studies are still needed to completely understand this link. Bioremediation using indigenous microbial communities already adapted to the contaminated environment thus appears to be a promising approach in the transformation and removal of CPs and PCDD/Fs from the polluted environment. However, little research has been conducted revealing the potential of secondary plant metabolites in accelerating the removal of PCP and PCDD/Fs from soils; the widely known secondary compound hypothesis indicates that secondary plant metabolites can stimulate microbial metabolism of pollutants present in the environment [117,118], which has been demonstrated with related groups of pollutants such as PCBs [119,120,121,122]. To the best of our knowledge, however, this mechanism remains unresolved for CPs and PCDD/Fs.

Additionally, further studies are needed to clarify the mechanisms of biodegradation of PCDD/Fs and the microbial communities directly involved. More knowledge needs to be gained about the interactions between aerobic and anaerobic degrading microorganisms, which inhabit polluted environments. Studies elucidating further genetic, enzymatic and regulatory foundations of biodegradation mechanisms should also be a priority. Therefore, it is important to keep monitoring PCP and PCDD/F concentrations in the environment, as impurities can still be emitted during synthesis and through the use of certain organics, such as pesticides.

## Abbreviations

AHD	aromatic-hydrocarbon-degrading
AI	active ingredient
CMC	carboxymethylcellulose
CDD/Fs	chlorinated dibenzo-*p*-dioxins and dibenzofurans
CNP	2,4,6-trichlorophenyl-4′-nitrophenyl ether
CP	chlorophenol
CPs	chlorinated phenols
d.w.	dry weight
DCP	dichlorophenol
DCDD	dichlorinated dibenzo-*p*-dioxin
DCDF	dichlorinated dibenzofuran
DD	dibenzo-*p*-dioxin
DF	dibenzofuran
FBC	fed-batch composting
HeCDD	heptachlorinated dibenzo-*p*-dioxin
HeCDF	heptachlorinated dibenzofuran
HxCDD	hexachlorinated dibenzo-*p*-dioxin
HxCDF	hexachlorinated dibenzofuran
MCPs	monochlorinated phenols
MSW	municipal solid waste
OCDD	octachlorinated dibenzo-*p*-dioxin
OCDF	octachlorinated dibenzofuran
PCDD	polychlorinated dibenzo-*p*-dioxin
PCP	pentachlorophenol
PeCDD	pentachlorinated dibenzo-*p*-dioxin
PCDF	polychlorinated dibenzofuran
PeCDF	pentachlorinated dibenzofuran
RDases	reductive dehalogenases
TCA	tricarboxylic acid
TCDD	trichlorinated dibenzo-*p*-dioxin
TCDF	trichlorinated dibenzofuran
TCHQ	tetrachlorohydroquinone
TCP	trichlorophenol
TeCDD	tetrachlorinated dibenzo-*p*-dioxin
TeCDF	tetrachlorinated dibenzofuran
TeCP	tetrachlorophenol
TEQ	toxic equivalent
